# Anti-cancer effects of ginsenoside compound k on pediatric acute myeloid leukemia cells

**DOI:** 10.1186/1475-2867-13-24

**Published:** 2013-03-12

**Authors:** Yan Chen, Yajing Xu, Yan Zhu, Xiaolin Li

**Affiliations:** 1 Department of Hematology, Xiangya Hospital, Central South University, Changsha, Hunan, 410008, P.R. China

**Keywords:** Ginsenoside, Compound K, Pediatric acute myeloid leukemia

## Abstract

Pediatric acute myeloid leukemia (AML) is a heterogeneous disease and remains clinically challenging. Currently chemotherapies are frequently associated with treatment-related death and long-term side effects. Therefore, alternative approaches with lower toxicity are highly desired. Ginsenosides and metabolites are the main ingredients responsible for the multiple pharmaceutical functions of ginseng, which is one of the most commonly consumed herbal medicines world widely. In the present study, we demonstrated that compound K, a major ginsenoside metabolite, inhibited the growth of the clinically relevant pediatric AML cell lines in a time- and dose-dependent manner. This growth inhibitory effect was attributable to suppression of DNA synthesis during cell proliferation. Furthermore, we observed significant G1 cell cycle arrest and apoptosis induced by compound K. The induction of apoptosis was accompanied by DNA double strand breaks. Our findings suggest that as a low toxic natural reagent, compound K could be a potential drug for pediatric AML intervention and to improve the outcome of pediatric AML treatment.

## Introduction

Pediatric acute myeloid leukemia (AML) remains a therapeutically challenging disease, which accounts for 15-20% of all childhood acute leukemia but is responsible for more than half of disease-specific deaths in these patients [1]. The treatment and the patient supportive care have improved significantly over the past decades, resulting in long-term survival in up to 65% of children with AML [2]. However, current chemotherapy is associated with high frequency of treatment related deaths and long-term side effects [3-5]. In addition, 20-40% patients do not respond to chemotherapy initially, while in those responsive patients, approximately 50% diseases will eventually relapse [2,6]. Therefore, new therapies for pediatric AML patients are urgently needed, especially for those with primary refractory or relapsed diseases.

Ginseng root is one of the most commonly used herbal medicines in the United States and East Asia for its multi-pharmaceutical functions [7-9]. Intake of ginseng root was reported to associate with reduced risk of various types of cancer in a case–control study conducted in Korea [10]. Ginsenosides are considered the main active ingredients responsible for the pharmaceutical activities of ginseng root [11]. Ginsenosides belong to a family of steroidal saponins and the two major groups of ginsenosides include protopanaxadiol and protopanaxatriol [12]. Orally taken ginseng products are usually not decomposed by gastric juice or liver enzymes before getting into the large intestine, where ginsenosides are metabolized by bacteria through deglycolation [12]. This process cleaves the sugar moieties of the ginsenosides in a stepwise manner to produce intermediate metabolites and finally protopanaxadiol-aglycone and protopanaxatriol-aglycone from protopanaxadiol group and protopanaxatriol group, respectively [12].

**Figure 1:**
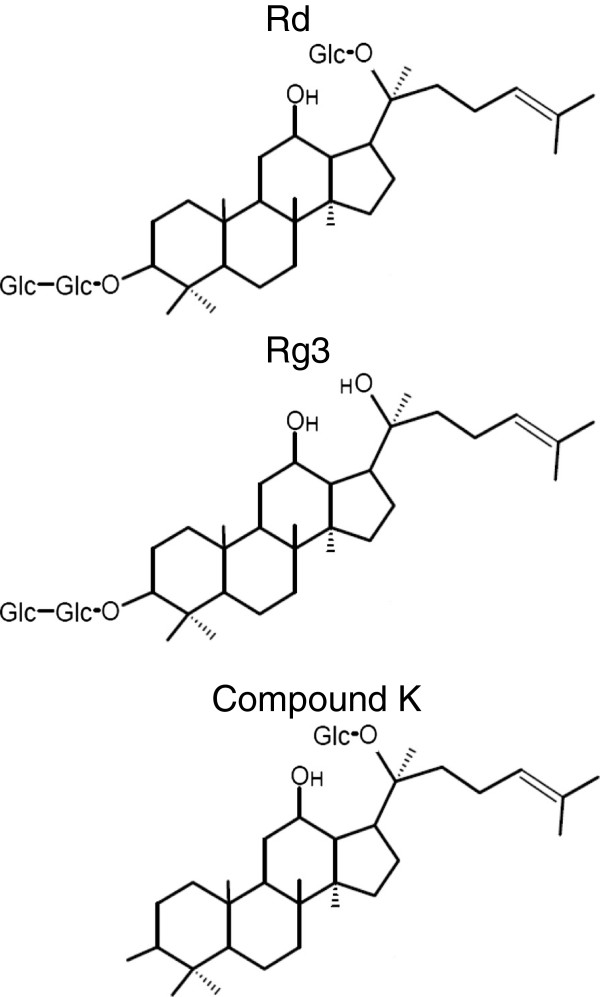
Structure of Rd, Rg3 and compound K.

Several ginsenosides have been reported to exert anticancer effects ascribed to their ability to inhibit DNA synthesis, angiogenesis and invasion, as well as induce cell cycle arrest and apoptosis [8]. For leukemia, ginsenoside Rh1 showed suppressive effect on MAPK signaling pathway, resulting inhibition of invasion and migration of THP-1 acute monocytic cells [13]. Another major intestinal ginsenoside metabolite compound K (structure shown in Figure 1) was shown to induce apoptosis through caspases-mediated pathways in HL-60 acute promyelocytic leukemia cells [14,15]. In this study, we describe a series of experiments designed to test the effect of compound K on pediatric acute myeloid leukemia cell lines and provide rationale for development of this ginsenoside as a potential therapeutic agent.

## Materials and methods

### Cell lines and reagents

The pediatric AML cell lines Kasumi-1 and MV4-11 were purchased from American Type Culture Collection (Manassas, VA). The cells were cultured in RPMI 1640 supplemented with 10% fetal bovine serum (FBS), plus 2 mM L-glutamine, 100 U/ml penicillin and 100 μg/ml streptomycin (Invitrogen). Compound K (99%) was purchased from the National Institute for the Control of Pharmaceutical and Biological Products (NICPBP, Beijing, China). All experiments were performed with the approved of the Central South University Institutional Care and Use Committee (IACUC).

### Cell viability assay

Cell viability was determined using the Sulforhodamine B (SRB) method as described [16]. Briefly, at the point of sample collection, the cells cultured in 96-well plates were fixed in 10% trichloroacetic acid (TCA) and incubated for 3 hr at 4°C. The fixed cells were washed with tap water for 4 times followed by staining with 0.4% SRB (Fluka) for 30 min at room temperature. The excess dye was washed off with 1% acetic acid for 4 times and the cells were dried by blow dryer. 150 μl 10 mM Tris-base (pH10.5) was added to each well to solubilize SRB by shaking the plates for 5 min on a shaker and the absorbance of SRB was measured at 550 nm.

### Cell proliferation assay

The cell proliferation was assayed based on the measurement of 5-bromo-2-deoxyuridine (BrdU) incorporated into cellular DNA during cell proliferation process by using the Cell Proliferation ELISA BrdU Kit (Roche Applied Science) as per manufacturer’s protocol. In brief, the BrdU labeling solution was added at the final concentration of 10 μM to the cell culture in 96-well plates during the last 4 hr of compound K treatment. After removal of labeling medium, FixDenat solution was added to the cells and incubated for 30 min at room temperature. The FixDenat solution was flicked off and the cells were incubated with anti-BrdU-POD solution for 90 min at room temperature. The cells were then washed with PBS for 3 times and incubated with Substrate solution for 15 min at room temperature, and the absorbance was measured at 405 nm (reference wavelength at 490 nm).

### Cell cycle analysis

Cells treated with compound K at indicated duration were trypsinized and fixed in 70% ethanol for ≥2 hr on ice. After a brief centrifugation, the cell pellet was suspended in propidium iodide staining solution containing 0.1% (v/v) Triton X-100, 0.2 mg/ml DNase-free RNase A, and 20 μg/ml propidium iodide. After incubation at room temperature for 30 min, cells were analyzed for DNA content by flow cytometry.

### Cell apoptosis assay

Cell apoptosis was detected based on assessing cytoplasmic histone-associated DNA fragments by using the Cell Death Detection ELISA^PLUS^ Kit (Roche Applied Science) as per manufacturer’s instruction. In brief, at the point of sample collection, detached cells in 96-well plates were precipitated by centrifugation and pooled with attached cells. Supernatant containing cytoplasmic fraction was transferred to the streptavidin-coated plate. Immunoreagent was then added to each well in the plate and incubated on a shaker for 2 hr at room temperature. The solution was removed and the plate was washed with incubation buffer for 3 times, followed by ABTS solution incubation until sufficient color was developed. The reaction was stopped by addition of ABTS Stop Solution and the absorbance was measured at 405 nm (reference wavelength at 492 nm).

### Western blotting

Soluble protein extracts or immunoprecipitated protein were boiled in sample buffer and subjected to SDS-polyacrylamide gel electrophoresis. Separated protein bands were electrophoretically transferred to polyvinylidene difluoride (PVDF) membrane (Thermal Fisher Inc.). Primary antibodies used were γH2AX, cleaved PARP, cleaved caspase-3 (Cell Signaling Technology, Cat. 9718, 5625 and 9664, respectively), and β–actin (Santa Cruz, sc-47778). The intensity of immunoreactive protein bands was measured using the Odyssey Infrared Imaging System (Li-Cor).

### Statistical analysis

Data are presented as the mean ± SEM from three independent experiments. The Student’s two-tailed t test was used to determine the difference between treatment group and control group.

## Results

### Compound K inhibits growth and proliferation of pediatric AML cells

**Figure 2:**
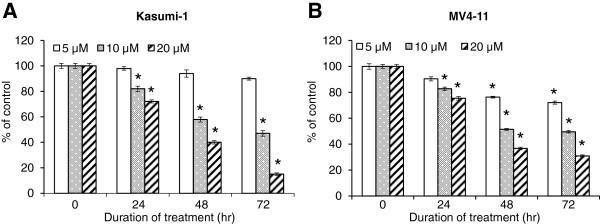
**Inhibitory effect of PPD on the growth of pediatric AML cell lines Kasumi-1 (A) and MV4-11 (B).** The cell viability was assessed by SRB assay following treatment with 5, 10 or 20 μM compound K for indicated duration. Bar: SEM. *: p < 0.05 from untreated control.

**Figure 3:**
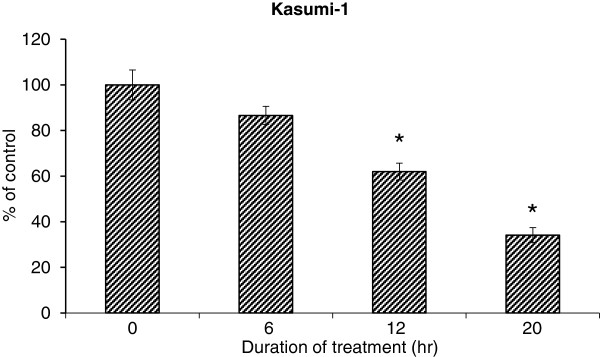
**Suppression of proliferation by compound K in Kasumi-1 cells.** Cell proliferation following treatment with 20 μM compound K for indicated duration was quantified by using colorimetric immunoassay based on measuring BrdU incorporation during DNA synthesis. Bar: SEM. *: p < 0.05 from untreated control.

We first examined the effect of compound K on the growth of Kasumi-1 and MV4-11 cells using the SRB assay as described in *Materials and Methods*. As presented in Figure 2A and 2B, the cells were treated with 5, 10 or 20 μM compound K for indicated time and compound K showed significant growth inhibition in a dose- and time-dependent manner in both cell lines. We then assessed the effect of compound K on Kasumi-1 cell proliferation as determined by DNA synthesis using the Cell Proliferation ELISA BrdU Kit. We found that the BrdU incorporation was significantly suppressed at 12 hr by 20 μM compound K, and this suppression became more dramatic at 20 hr of treatment (Figure 3). Notably, the inhibition of proliferation at 20 hr was already ~65%, while the inhibition of growth at 24 hr was only ~30%, indicating that the effect of compound K on proliferation is not likely a secondary result of growth inhibition.

### Compound K induces cell cycle arrest and apoptosis of pediatric AML cells

**Figure 4:**
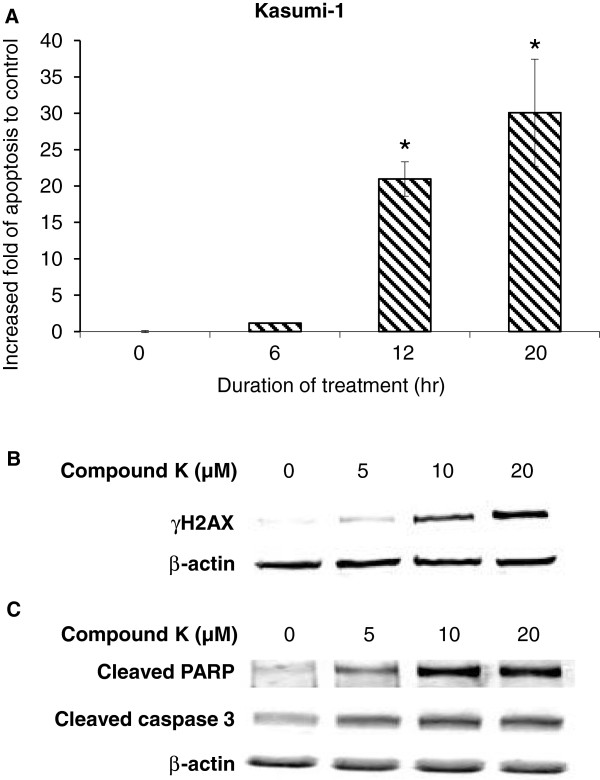
**Induction of apoptosis by compound K in Kasumi**-**1 cells. A**. Cell apoptosis following treatment with 20 μM compound K for indicated duration was quantified by using photometric enzyme-immunoassay based on measuring cytoplasmic histone-associated-DNA-fragmentation. Column: fold of induced apoptosis index compared to untreated control. Bar: SEM. *: p < 0.05. **B** &**C**. Western blot analysis of γH2AX, cleaved PARP and cleaved caspase-3. Kasumi-1 cells were treated with different doses of compound K as indicated for 20 hr and then subjected to Western blot analysis.

In order to further demonstrate the effects of compound K on earlier cellular events, we then evaluated Kasumi-1 cell cycle distribution upon compound K treatment. The cells were treated with 20 μM compound K for indicated duration and then processed and subjected to flow cytometry analysis. The percentage of DNA content at different cell cycle phases was presented in Table 1, showing that starting from 12 hr of compound K treatment, significant G1 arrest was observed. The effect continued to escalate with time. We next examined the effect of compound K on apoptosis in Kasumi-1 cells by using the Cell Death Detection ELISA^PLUS^ Kit that quantitatively determines cytoplasmic histone-associated DNA fragments induced by cell death. We found that compound K induced significant apoptosis at 12 hr of treatment and this induction became more evident at 20 hr (Figure 4A). Compound K-induced apoptosis was further validated by Western blotting analysis of γH2AX, the phosphorylated form of H2AX as the cellular response to DNA double strand breaks [17]. As shown in Figure 4B, compound K treatment led to increased expression of γH2AX in a dose-dependent manner. In support of this finding, the expression of cleaved PARP and caspase-3 was also significantly elevated in response to compound K treatment (Figure 4C), suggesting compound K-induced DNA double strand breaks triggered apoptosis. This is consistent with our cell growth inhibition result. Taken together, these observations suggest that the growth inhibitory effect of compound K on the pediatric AML cells could be attributable to suppression of cell proliferation and induction of G1 cell cycle arrest and apoptosis.

**Table 1:** **Compound K induces G1 cell cycle arrest of Kasumi**-**1**

	**G1**	**S**	**G2**
0 hr	74.58 ± 1.38	17.49 ± 1.66	9.48 ± 0.29
6 hr	72.24 ± 2.54	18.29 ± 1.58	9.90 ± 0.86
12 hr	79.15 ± 1.12*	11.56 ± 0.36*	9.15 ± 0.63
20 hr	83.25 ± 1.69*	8.36 ± 0.74*	8.80 ± 0.87

Induction of G1 cell cycle arrest by compound K in Kasumi-1 cells. Cell cycle distribution was determined by flow cytometry analysis. Data represent % of cells at different cell cycle phases. *: p < 0.05 from untreated control.

## Discussion

Improvements in supportive care, including the use of antibiotics, antifungal treatment, matched related in bone marrow transplantation, increased days of conventional induction chemotherapy, have contributed significantly to the prolonged survival of patients with pediatric acute myeloid leukemia (AML) [2,3]. However, further intensification of antileukemia therapies may cause treatment-related long-term side effects and mortality [2,3]. Therefore, it is imperative to develop novel approaches with lower toxicity to improve the outcome of pediatric AML treatment.

The use of ginseng has been documented for centuries in the Far East, especially in China and Korea, for improving physical vitality and curing diseases [18]. Nowadays, ginseng and its products are among the most commonly consumed herbal medicines as a tonic for enhancing well-being and also as a complementary therapy for cancer intervention in many countries [7]. Common side effects caused by overdose of ginseng include inability to sleep, nausea, diarrhea, epistaxis, high blood pressure and headache [19]. However, ginseng is very well tolerated by most people and considered nontoxic at high dose to the animals tested, making it an attractive candidate for disease intervention [20].

As the main ingredients, over 100 ginsenosides have been identified and some of them have been tested in several cancer models including leukemia [21]. Interestingly, it appears that the sugar chain complex is inversely associated with the anti-cancer effect of ginsenosides [12]. The proposed mechanism mediating this inverse association is that ginsenosides with less sugar moieties have increased hydrophobic character, which lead to increased permeability of cell membrane and therefore the uptake of compounds [22]. As a major intermediate metabolite of ginsenosides, compound K has relatively short side chain compared with its precursors (Figure 1) [12], and has been shown to induce apoptosis in human leukemia cells [14]. In the present study, we demonstrated for the first time the anti-cancer effect of compound K in a clinically relevant cell line, Kasumi-1 [23]. In addition to cell growth inhibition, compound K suppresses cell DNA synthesis and induces cell cycle arrest at G1 phase. Furthermore, we showed that compound K was able to induce DNA double strand breaks associated with apoptosis. Our findings suggest that as a natural existing, low toxicity reagent, compound K is a potential approach for pediatric AML therapy. Future study may focus on combination of compound K with conventional chemotherapies to lower the dose and improve the outcome of pediatric AML treatment.

## Competing interests

None of the authors of this study has a conflict of interest statement.

## Authors’ contribution

YC, YX and YZ performed experiments and collected data. YC and XL designed this study and drafted the article. All authors read and approved the final manuscript.
